# The development of contemporary European sea bass larvae (*Dicentrarchus labrax*) is not affected by projected ocean acidification scenarios

**DOI:** 10.1007/s00227-017-3178-x

**Published:** 2017-06-29

**Authors:** Amélie Crespel, José-Luis Zambonino-Infante, David Mazurais, George Koumoundouros, Stefanos Fragkoulis, Patrick Quazuguel, Christine Huelvan, Laurianne Madec, Arianna Servili, Guy Claireaux

**Affiliations:** 1 Ifremer, Laboratoire Adaptation, Reproduction et Nutrition des poissons, LEMAR (UMR 6539), 29280 Plouzané, France; 2 Université de Bretagne Occidentale, LEMAR, (UMR 6539), Laboratoire Adaptation, Reproduction et Nutrition des poissons, 29280 Plouzané, France; 3 Biology Department, University of Crete, Vasilika Vouton, 70013 Heraklio, Crete Greece

## Abstract

Ocean acidification is a recognized consequence of anthropogenic carbon dioxide (CO_2_) emission in the atmosphere. Despite its threat to marine ecosystems, little is presently known about the capacity for fish to respond efficiently to this acidification. In adult fish, acid–base regulatory capacities are believed to be relatively competent to respond to hypercapnic conditions. However, fish in early life stage could be particularly sensitive to environmental factors as organs and important physiological functions become progressively operational during this period. In this study, the response of European sea bass (*Dicentrarchus labrax*) larvae reared under three ocean acidification scenarios, i.e., control (present condition, $P_{{{\text{CO}}_{2} }}$ = 590 µatm, pH total = 7.9), low acidification (intermediate IPCC scenario, $P_{{{\text{CO}}_{2} }}$ = 980 µatm, pH total = 7.7), and high acidification (most severe IPCC scenario, $P_{{{\text{CO}}_{2} }}$ = 1520 µatm, pH total = 7.5) were compared across multiple levels of biological organizations. From 2 to 45 days-post-hatching, the chronic exposure to the different scenarios had limited influence on the survival and growth of the larvae (in the low acidification condition only) and had no apparent effect on the digestive developmental processes. The high acidification condition induced both faster mineralization and reduction in skeletal deformities. Global (microarray) and targeted (qPCR) analysis of transcript levels in whole larvae did not reveal any significant changes in gene expression across tested acidification conditions. Overall, this study suggests that contemporary sea bass larvae are already capable of coping with projected acidification conditions without having to mobilize specific defense mechanisms.

**Electronic supplementary material:**

The online version of this article (doi:10.1007/s00227-017-3178-x) contains supplementary material, which is available to authorized users.

## Introduction

Over the last two centuries, the intensification of human activities has led to a rise in the atmospheric concentration of carbon dioxide. Nowadays, this concentration is in excess of 400 ppm, a level which has never been reached in the past 800,000 years (Lüthi et al. 2008). At the current rate of emissions, atmospheric CO_2_ concentration is projected to reach between 750 and 1000 ppm by the end of the twenty-first century (Intergovernmental Panel on Climate Change 2014). More severe scenarios even project an increase up to 1500 ppm (Caldeira and Wickett 2005). Atmospheric CO_2_ is absorbed by the oceans where it reacts to form carbonic acid (H_2_CO_3_) which then dissociates into bicarbonate (HCO_3_^−^) and hydrogen (H^+^) ions. The increase in H^+^ concentration reduces the ocean pH, which represents the widely recognized phenomenon called “ocean acidification.” Ocean surface pH has been reduced by 0.1 pH unit (U) during the last century and an additional drop of 0.3–0.5 U is expected by the end of the present century (Caldeira and Wickett 2005; Intergovernmental Panel on Climate Change 2014).

It is generally accepted that predicted conditions of ocean acidification, along with the decline of marine carbonate ion saturation states, will have deleterious effects on calcifying organisms such as corals, coralline algae, molluscs, and echinoderms (reviewed in Orr et al. 2005; Melzner et al. 2009; Kroeker et al. 2010). Surprisingly, little is known about the capacity of internally calcifying marine organisms, and especially fish, to respond to ocean acidification. The mechanistic bases of acid–base regulation are relatively well described in fish, and they are recognized to be particularly efficient to respond to hypercapnic conditions (reviewed in Pörtner et al. 2004; Ishimatsu et al. 2008; Heuer and Grosell 2014). However, a review of the literature shows that most of the available reports describe short-term responses to hypercapnia (hours to few days) and mostly in adult and juvenile fish. Studies on long-term exposure to projected ocean acidification levels and on fish in early life stages are relatively rare.

Fish embryonic and larval stages are considered sensitive to environmental factors and particularly to ambient pH (Baumann et al. 2012; Forsgren et al. 2013; Tseng et al. 2013; Frommel et al. 2014). During these life stages, organs and important physiological functions progressively become operational (reviewed in Zambonino-Infante and Cahu 2001; Ishimatsu et al. 2008). These include, among others: the gut (reviewed in Zambonino-Infante and Cahu 2001); the gill (Varsamos et al. 2002); the skin (Padros et al. 2011); and the urinary system (Nebel et al. 2005). All of these functions are involved in acid–base regulation (reviewed in Varsamos et al. 2005). A perturbation of the development of the different organs and functions during that period is liable to have long-lasting effects on animal performance and fitness (Boglione et al. 2013; Vanderplancke et al. 2015) and, therefore, influence population resilience (reviewed in Houde 2008).

Previous studies have reported that larvae functional skeletal and gut developments can be influenced by environmental conditions including the presence of pollutants, reduced water oxygen level (hypoxia), and inappropriate nutrition or temperature (Boglione et al. 2013; Vanderplancke et al. 2015). Regarding water acidification, recent studies have highlighted an effect on a wide range of larval physiological (acid–base regulation, metabolism, tissue and growth damages) and behavioral (sensory and predator–prey interaction) processes (Melzner et al. 2009; Forsgren et al. 2013; Frommel et al. 2014; Pimentel et al. 2014). However, research on the influence of ocean acidification on the functional development of fish larvae is still scarce. The few studies that explored these effects revealed no influence on skeletal development and only limited effect on gut maturation (Munday et al. 2011a; Pimentel et al. 2015).

A molecular holistic and integrative view of the physiological processes involved in fish response to water acidification is also crucially lacking. So far, studies looking the effect of ocean acidification on fish mainly focused on specific regulatory processes, while a multifunctional approach would be desirable to highlight possible functional trade-off. Few studies have implemented global approaches, without a priori hypotheses, to investigate the mechanisms underlying fish response (Maneja et al. 2014; Schunter et al. 2016) and these essentially focussed on the proteomic level or on tissue-specific response.

In the present study, the larval response to ocean acidification was thus investigated in a temperate fish species, the European sea bass (*Dicentrarchus labrax*), by assessing larvae performance across multiple levels of biological organization. More specifically, the objectives were (1) to document the survival and growth of the larvae as proxies of whole organism response to expected acidification conditions; (2) to evaluate the structural development of the larvae by investigating skeleton ossification; (3) to examine their physiological development through analyzing the maturation of their digestive functions; and (4) to examine the molecular mechanisms underlying larval response by combining global (microarray analysis) and targeted (qPCR) approaches.

## Materials and methods

### Animals and experimental conditions

Fish breeding was performed at a local commercial fish farm (Aquastream, Lorient, France) in October 2013. Breeders, derived from a population domesticated for five generations, were used in a factorial breeding design (three blocks of four dams crossed reciprocally to five to seven sires, i.e., 20 to 28 crosses by block) to produce 76 families. The fertilized eggs were all mixed and reared under similar conditions (pH about 7.9 ± 0.1) until two days-post-hatching (dph). The larvae were then transferred to Ifremer rearing facility in Brest (France) and randomly distributed among three experimental conditions. These conditions were established on the basis of the projected partial pressure of CO_2_ ($P_{{{\text{CO}}_{2} }}$) scenarios for 2100 (Intergovernmental Panel on Climate Change 2014), i.e., control (labeled C); present condition, ($P_{{{\text{CO}}_{2} }}$ = 590 µatm, pH total = 7.9); low acidification (LA; intermediate IPCC scenario, $P_{{{\text{CO}}_{2} }}$ = 980 µatm, pH total = 7.7); and high acidification (HA; the most severe IPCC scenario, $P_{{{\text{CO}}_{2} }}$ = 1520 µatm, pH total = 7.5). In each condition, three replicate 38-L flow-through tanks were used, with approximately 2200 larvae per tank *(n* = 6600 larvae for each experimental condition). Water temperature was maintained at 19 °C, oxygen concentration above 90%, salinity at 34‰ and the photoperiod was set at 24D during the first week and then at 16L:8D. Larvae were fed ad libitum via a continuous delivery of *Artemia* until 28 dph and then by a continuous automated supply of commercial dry pellets (Néo-start, Le Gouessant Aquaculture, France) until 45 dph.

Tanks were supplied through-flowing seawater pumped at 500 m from the coast line and from a depth of 20 m. The water was then filtered through a sand filter and passed successively through a tungsten heater, a degassing column packed with plastic rings, a 2-µm filter membrane, and a UV lamp. These steps guaranteed high-quality seawater. Each set of triplicate tanks was equipped with a header tank (100 L) which was used to control the pH of the water. Each header tank was equipped with an automatic injection system connected to a pH electrode (pH Control, JBL, Germany) which injected either air (control) or CO_2_ (treatments). A daily measurement of water temperature and pHNBS in the fish tanks was taken with a handheld pH meter (330i, WTW, Germany) calibrated weekly with fresh buffers (Merk, Germany). Measured values never differed by more than 2% from the target values. In addition, at the beginning and at the end of the larvae rearing phase, the total pH was determined following Dickson et al. (2007) using m-cresol purple as the indicator. Total alkalinity (TA) in each experimental condition was also measured at the beginning and at the end of the larvae rearing, by titration (LABOCEA, France). Phosphate and silicate concentrations were determined by segmented flow analysis following Aminot et al. (2009). $P_{{{\text{CO}}_{2} }}$ was calculated using CO_2_SYS software (Lewis and Wallace 1998) and constants from Mehrbach et al. (1973). Water chemistry is summarized in Table 1.

**Table 1:** Water chemistry of the experimental conditions

Conditions	Salinity (‰), *n* = 2	T °C, *n* = 42	pH_NBS_, *n* = 42	pH total, *n* = 2	TA (µmol L^−1^), *n* = 2	PO_4_^3−^ (µmol L^−1^), *n* = 2	SiO_4_ (µmol L^−1^), *n* = 2	$P_{{{\text{CO}}_{2} }}$ (µatm), *n* = 2
Control	33.8 ± 0.2	19.2 ± 0.3	7.96 ± 0.01	7.89 ± 0.01	2294 ± 3	0.57 ± 0.01	8.94 ± 0.06	589 ± 10
LA 7.7	33.8 ± 0.2	19.2 ± 0.3	7.79 ± 0.01	7.71 ± 0.05	2298 ± 1	0.57 ± 0.01	8.94 ± 0.06	978 ± 41
HA 7.5	33.8 ± 0.2	19.2 ± 0.3	7.59 ± 0.01	7.54 ± 0.03	2306 ± 9	0.57 ± 0.01	8.94 ± 0.06	1521 ± 97

T °C represents the water temperature, TA the total alkalinity, PO_4_^3−^ the phosphate concentration, SiO_4_ the silicate concentrations, and $P_{{{\text{CO}}_{2} }}$ the projected partial pressure of CO_2_

Mean ± SEM; *n* is the number of samples

### Sampling

Starting at 15 dph, 10 larvae per tanks (*n* = 30 larvae per experimental condition) were sampled weekly, anaesthetized with phenoxyethanol (200 ppm), and their body wet mass (to the nearest 0.01 mg) was measured. After 28 dph, their fork length (± 0.01 mm) was also measured. At 45 dph, a total of 120 larvae per tank were sampled. Thirty of these larvae were used to measure body mass and fork length. Twenty larvae per tank were anaesthetized with phenoxyethanol and placed in 5% formalin for later ossification and skeletal malformation analysis. Three pools of 20 larvae per tank were anaesthetized with phenoxyethanol (200 ppm), rapidly frozen in liquid nitrogen and stored at −80 °C until enzymatic analysis. Finally, 10 larvae per tank were anaesthetized with phenoxyethanol and stored in RNA later until transcriptomic analysis. The total number of larvae remaining in each tank at 45 dph was also counted and the relative survival determined.

### Skeletal analysis

The formalin fixed larvae were bleached with KOH and peroxide treatment and then double stained with alcian blue for cartilaginous structures and alizarin red S for ossified structures (Darias et al. 2010a). Larvae from the different experimental conditions were stained in the same batch to reduce variability, and they were stored in 100% glycerol. Stained larvae were placed on Petri dishes and scanned (Epson Perfection 4990 Photo, Epson America, Long Beach, CA, USA) to create a digital image with a resolution of 3200 dpi. Image analysis of the ossified larval surface was carried out as described in Darias et al. (2010a). A calcification index representing the degree of bone mineralization was then calculated for each larva as a ratio of mineralized bones to cartilage components. The study of skeletal malformation focused on macroscopically evident skeletal deformities and on internal deformities of moderate severity. The examination of the malformations was carried out randomly by experienced examiners using coded images. The macroscopically evident deformities encompassed externally visible deformities (i.e., deformities of the vertebral column such as lordosis, scoliosis or kyphosis and of the mouth such as prominent or retracted lower jaw) in addition to the incidence of vertebral compression or fusion with no apparent body distortion. Internal deformities of moderate severity encompassed jaw deformities (such as the abnormal convergence of the two dentaries in lower jaw, and abnormal anatomy of upper jaw), fin deformities (abnormal anatomy of the fin supporting elements), and occurrence of urinary calculi.

### Enzymatic analysis

Larvae were dissected as described in Cahu and Zambonino-Infante (1994) to separate pancreatic and stomach segments from the intestinal segment. The dissected segments were homogenized in five volumes of cold distilled water. Trypsin and amylase were assayed according to Holm et al. (1988) and Métais and Bieth (1968), respectively. Brush border membranes were purified from the intestinal segment homogenate according to a method developed by Crane et al. (1979) and modified by Zambonino-Infante et al. (1997). The cytosolic enzyme, leucine–alanine peptidase, was assayed using the method of Nicholson and Kim (1975). Enzymes of the brush border membranes, alkaline phosphatase and aminopeptidase N were assayed according to Bessey et al. (1946) and Maroux et al. (1973), respectively. Protein concentration was determined according to Bradford (1976). Enzymatic analyses were performed according the recommendations of the authors’ methods with blanks. Only endpoint techniques (amylase and leucine–alanine peptidase) used calibration curves. Other enzyme determinations were based on kinetic measures.

Pancreatic enzymes activity (trypsin and amylase) was expressed as a ratio of activity in the intestinal segment to that in both the pancreatic and intestinal segments. Activity of intestinal enzymes was expressed as a ratio of alkaline phosphatase to leucine–aminopeptidase activity in the brush border membranes to the activity of the cytosolic enzyme leucine–alanine peptidase.

### Transcriptomic analysis

Larvae were homogenized individually, and total RNA was extracted as previously described (Vanderplancke et al. 2015).

*Microarray analysis*: Transcription profiles of five samples per tanks (i.e., 15 samples per experimental condition, 45 samples in total) were performed using 44 K whole sea bass genome microarrays (Agilent) that contained 38,130 probes, providing high coverage of sea bass transcripts. Double-stranded cDNA was synthesized from 500 ng of total RNA using the Quick Amp Labeling kit, One color (Agilent). Labeling with cyanine 3-CTP, fragmentation of cRNA, hybridization, and washing were performed according to the manufacturer’s instructions (Agilent) at the Laboratoire de Génétique Moléculaire et d’Histocompatibilité (Brest, France). The microarrays were then scanned and the data extracted with the Agilent Feature Extraction Software.

*qPCR analysis:* Total RNA of the 30 samples per condition was reverse-transcribed into cDNA using iScript™ cDNA Synthesis kit (Bio-Rad Laboratories, Hercules, CA, USA). Quantitative real-time polymerase chain reaction (qPCR) was used to specifically analyze seven candidate genes: osteocalcin, which are implied in osteoblast differentiation and mineralization (Lein and Stein 1995); carbonic anhydrase 2 (CA2) and carbonic anhydrase 4 (CA4), which contribute to CO_2_ hydration; NA^+^/H^+^ exchanger 1 (Slc9a1), NA^+^/H^+^ exchanger 3 (Slc9a3), NA^+^/HCO_3_^−^ co-transporter (Slc4a4), and HCO_3_^−^ transporters (Slc4a1), which are involved in dynamic adjustments of acid–base balance (reviewed in Heuer and Grosell 2014). The design of specific primers for each gene was performed using Primer3Plus software based on cDNA sequences available in public databases NCBI (https://www.ncbi.nlm.nih.gov) or SIGENAE (http://www.sigenae.org/) (Table 2). The qPCR for each gene was performed using iQ™ SYBR^®^ Green Supermix (Bio-Rad Laboratories, Hercule, USA) as previously described (Vanderplancke et al. 2015). Glyceraldehyde-3-phosphate dehydrogenase (GAPDH) gene was chosen as the housekeeping gene as it showed minimal variance in its transcript level, both within and between conditions.

**Table 2:** Oligonucleotide primers used for quantitative PCR. § refers to NCBI database (https://www.ncbi.nlm.nih.gov) and $ to SIGENAE database (http://www.sigenae.org/)

Gene name	Abbreviation	Accession number	Species	Forward (F) and reverse (R) primers sequences (5′–3′)
Osteocalcin	BGLAP	AY663813 (§)	*Dicentrarchus labrax*	F: ATGGACACGCAGGGAATCATTG
				R: TGAGCCATGTGTGGTTTGGCTT
Carbonic anhydrase 2	CA2	FK944087.p.dl.5 ($)	*Dicentrarchus labrax*	F: CTGATACATGGGGAGCCGAT
				R: AAAGAGGAGTCGTACTGGGC
Carbonic anhydrase 4	CA4	CX660749.p.dl.5 ($)	*Dicentrarchus labrax*	F: ACCTTTCAGAACTACGGCGA
				R: TGGAACTGCAGGCTGTCATA
NA^+^/H^+^ exchanger 1	Slc9a1	EU180587 (§)	*Dicentrarchus labrax*	F: GGATGCTGGCTACTTTCTGC
				R: GGATTGCCTGGCTGAATCTG
NA^+^/H^+^ exchanger 3	Slc9a3	CX660524.p.dl.5 ($)	*Dicentrarchus labrax*	F: CCTCTAACGGCCTCATACCA
				R: CCTGACATCATGGCTGACAC
NA^+^/HCO_3_^−^ co-transporter	Slc4a4	FM001880.p.dl.5 ($)	*Dicentrarchus labrax*	F: CAGATCTGCCAGTAAACGCC
				R: AAAGCCACATGTCTCTCCGA
HCO_3_^−^ transporters	Slc4a1	AM986338.p.dl.5 ($)	*Dicentrarchus labrax*	F: TACCAGCATTCAGGGTGTCA
				R: TTCCTCAAACACCAGCAGTG

### Statistical analysis

Data normality and homogeneity of all variances were tested with Kolmogorov–Smirnov and Brown–Forsythe tests, respectively. The macroscopically evident deformities index had to be rank transformed to obtain normality (Quinn and Keough 2002). Body mass and fork length were then analyzed using mixed model:

$$yijk = \mu + {\text{AS}}i + {\text{AC}}j + ({\text{AS}} \times {\text{AC}})ij + {\text{T}}jk + {\text{e}}ijk,$$

where *yijk* is the phenotypic observation, *μ* is the sample mean, AS*i* is the effect of the *i*th age stage, and AC*j* is the effect of the *j*th acidification condition, which were all fitted as fixed effects as well as their interactions; T*jk* is the effect of the *k*th tank nested in *j*th acidification condition fitted as a random effect, and *eijk* is the random residual effect. The percentage of larvae survival at 45 dph was analyzed using one-way ANOVA with the acidification condition as factor. The calcification ratio was analyzed using ANCOVAs with the acidification condition fitted as a fixed effect, the tank effect nested in the acidification condition fitted as a random effect, and the fork length as the co-variable. Other variables (i.e., percentage of deformities, enzymatic capacities, enzymatic ratios and genes expression profiles) were analyzed using the mixed model with the acidification condition fitted as a fixed effect and the tank effect nested in the acidification condition fitted as a random effect. The a posteriori Tukey’s honest significant difference (HSD) tests were used for mean comparisons when possible or replaced by Games and Howell tests when variances were not homogenous. Analyses were carried out using Statistica version 7.0 for windows (StatSoft, USA). The microarray expression data were analyzed at the BIOSIT Health Genomic core facility (Rennes, France). Data were normalized using Agilent *Genespring GX* software, and statistical analysis was performed with *FactoMineR* and *nlme* R packages using mixed model with the acidification condition as a fixed effect and the tank as a random effect. The *P* values were then corrected for multiple testing using Benjamini–Hochberg threshold. A significance level of *α* = 0.05 was used in all statistical tests.

## Results

### Survival and growth

Larval survival rate was in excess of 50%. However, significant differences were observed among experimental conditions, with fish reared under low acidification (LA) exhibiting a survival rate of 21.5 ± 5.5% higher than those reared in the two other conditions (Table 3).

**Table 3:** Growth performance, measured as body mass (mg) and length (mm), and survival (%) of the sea bass larvae during the rearing at different days-post-hatching (dph) in the three different experimental conditions: control condition ($P_{{{\text{CO}}_{2} }}$ = 590 µatm, pH total = 7.9), low acidification condition (LA 7.7, $P_{{{\text{CO}}_{2} }}$ = 980 µatm, pH total = 7.7), and high acidification condition (HA 7.5, $P_{{{\text{CO}}_{2} }}$ = 1520 µatm, pH total = 7.5)

Age	Condition	*n*	Mass (mg)	Length (mm)	*n*	Survival (%)
15 dph	Control	30	2.14 ± 0.08			
	LA 7.7	30	1.98 ± 0.06			
	HA 7.5	30	1.94 ± 0.06			
21 dph	Control	30	5.65 ± 0.35			
	LA 7.7	30	5.31 ± 0.32			
	HA 7.5	30	6.52 ± 0.34			
28 dph	Control	30	12.74 ± 1.04	12.40 ± 0.31		
	LA 7.7	30	9.20 ± 0.93	11.43 ± 0.30		
	HA 7.5	29	10.29 ± 0.96	11.91 ± 0.31		
35 dph	Control	30	27.43 ± 1.87	14.87 ± 0.30^b^		
	LA 7.7	30	22.64 ± 1.38	13.88 ± 0.23^a^		
	HA 7.5	30	26.48 ± 1.11	14.84 ± 0.15^b^		
45 dph	Control	90	57.80 ± 1.90^b^	18.96 ± 0.20^b^	3	48.56 ± 2.82^a^
	LA 7.7	90	49.88 ± 1.95^a^	17.94 ± 0.23^a^	3	61.67 ± 1.78^b^
	HA 7.5	90	63.75 ± 1.91^b^	19.54 ± 0.19^b^	3	53.15 ± 1.13^a^

Mean ± SEM; *n* is the number of individuals

The different letters indicate significant differences among conditions (*α* = 0.05)

Experimental conditions significantly influenced larval growth, and a significant interaction with time was found [ANOVA, *F*(8,614) = 3.37, *P* < 0.001; Table 2]. Growth performance (mass and length) was similar among conditions until 35 dph (Table 3). From that time, fish from the LA group were significantly 6.7 ± 0.8% shorter than fish reared in the two other conditions. At 45 dph, fish from LA also displayed significantly 17.7 ± 4.0% lower mass than fish reared in the two other conditions (Table 3).

### Skeletal development

**Fig. 1:**
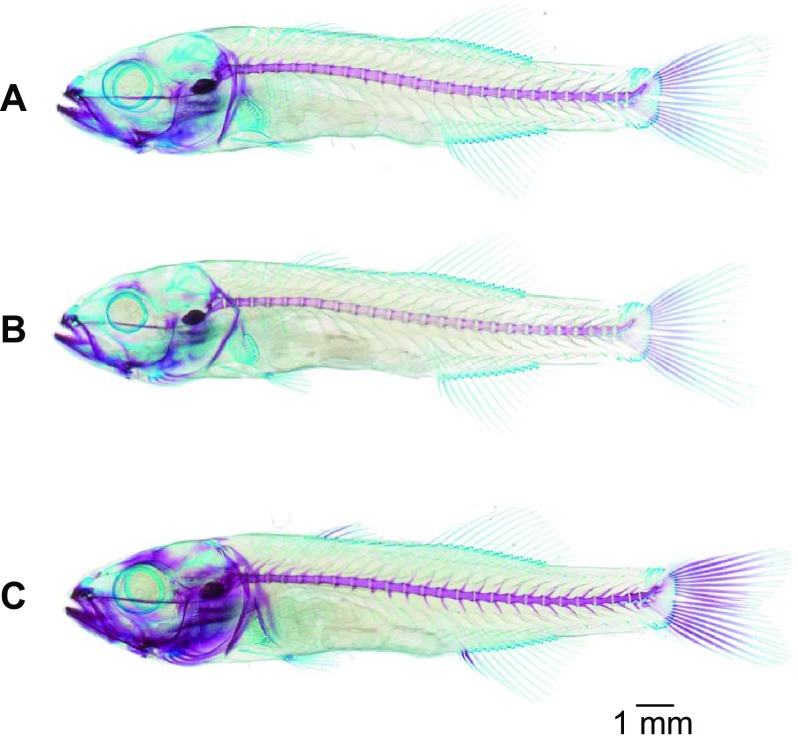
Representation of the calcification of the double-stained 45 days-post-hatching larvae according to the three experimental conditions **a** control condition ($P_{{{\text{CO}}_{2} }}$ = 590 µatm, pH total = 7.9); **b** low acidification condition (LA 7.7, $P_{{{\text{CO}}_{2} }}$ = 980 µatm, pH total = 7.7); **c** high acidification condition (HA 7.5, $P_{{{\text{CO}}_{2} }}$ = 1520 µatm, pH total = 7.5). The three individuals are representatives to their experimental conditions in calcification and size. Mineralized bones are in *red* and cartilages components in *blue*

**Fig. 2:**
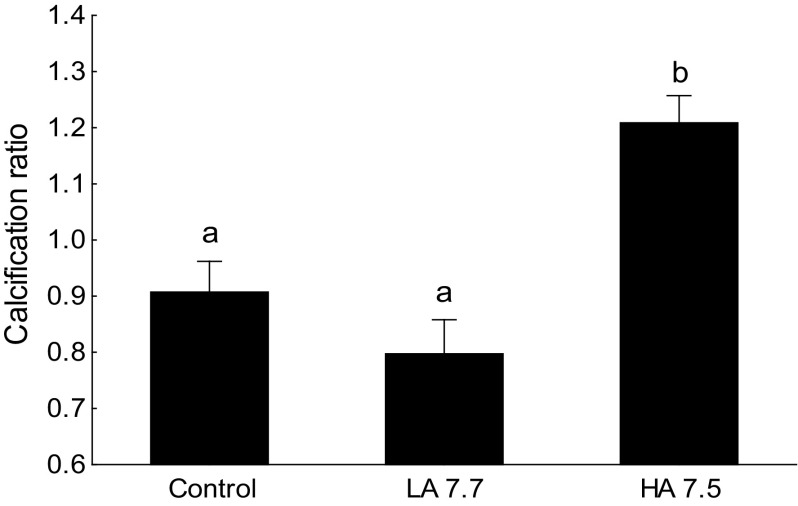
Calcification ratio, relative proportion of mineralized bones vs. cartilages components, of the 45 days-post-hatching sea bass larvae in the three different experimental conditions: control condition ($P_{{{\text{CO}}_{2} }}$ = 590 µatm, pH total = 7.9), low acidification condition (LA 7.7, $P_{{{\text{CO}}_{2} }}$ = 980 µatm, pH total = 7.7), and high acidification condition (HA 7.5, $P_{{{\text{CO}}_{2} }}$ = 1520 µatm, pH total = 7.5). Higher values suggest larger proportion of mineralized bones. Mean ± SEM; *n* = 60 per experimental conditions. The *different letters* indicate significant differences among conditions (*α* = 0.05)

A significant impact of the experimental conditions on the calcification ratio was observed [ANCOVA, *F*(2,177) = 10.97, *P* < 0.001]. At 45 dph, this ratio was significantly higher in the larvae from the HA condition (7.6) than in the two other conditions, indicating that HA larvae had more mineralized bones than larvae from the other conditions (Figs. 1, 2).

**Fig. 3:**
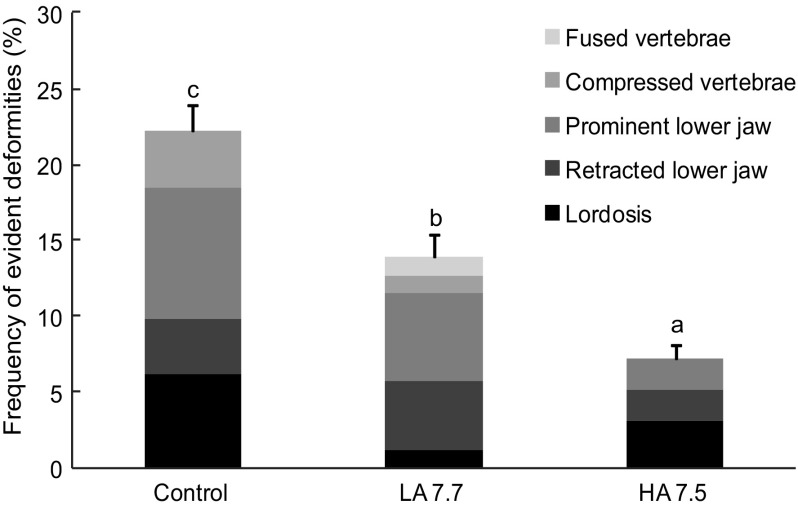
Frequency (%) of macroscopically evident deformities of 45 days-post-hatching larvae in the three different experimental conditions: control condition ($P_{{{\text{CO}}_{2} }}$ = 590 µatm, pH total = 7.9), low acidification condition (LA 7.7, $P_{{{\text{CO}}_{2} }}$ = 980 µatm, pH total = 7.7), and high acidification condition (HA 7.5, $P_{{{\text{CO}}_{2} }}$ = 1520 µatm, pH total = 7.5). The macroscopically evident deformities encompassed deformities of the vertebral column (lordosis), of the mouth (prominent or retracted lower jaw), and vertebral compression or fusion. Mean ± SEM. The *different letters* indicate significant differences among conditions (*α* = 0.05)

The frequency of macroscopically evident deformities was also significantly influenced by the experimental conditions [ANOVA, *F*(2,51) = 30.54, *P* < 0.001]. Larvae from HA had significantly lower occurrence of macroscopically evident deformities (lordosis, prominent or retracted lower jaw, compressed or fused vertebrae) than larvae from the other two experimental conditions (Fig. 3). Experimental conditions also had a significant effect on the occurrence of abnormal convergence of the two dentaries in the lower jaw [ANOVA, *F*(2,9) = 5.82, *P* = 0.04], with the HA larvae displaying significantly lower occurrence (Table 4). On the other hand, experimental conditions did not influence the frequency of upper jaw, anterior dorsal fin, posterior dorsal fin, anal fin, and caudal fin deformities, neither the occurrence of urinary calculi [ANOVAs, *F*(2,9) = 1.85, *P* = 0.23; *F*(2,9) = 1, *P* = 0.42; *F*(2,9) = 1.21, *P* = 0.36; *F*(2,9) = 0.06; *P* = 0.94; *F*(2,9) = 1.02, *P* = 0.41; *F*(2,9) = 2.25*, P* = 0.18, respectively, Table 4].

**Table 4:** Frequencies (%) of the internal deformities of light severity of the 45 days-post-hatching larvae in the three different experimental conditions: control condition ($P_{{{\text{CO}}_{2} }}$ = 590 µatm, pH total = 7.9), low acidification condition (LA 7.7, $P_{{{\text{CO}}_{2} }}$ = 980 µatm, pH total = 7.8), and high acidification condition (HA 7.5, $P_{{{\text{CO}}_{2} }}$ = 1520 µatm, pH total = 7.6). The internal deformities of light severity encompassed deformities of the jaw (lower and upper jaw), of the fin (anterior dorsal, posterior dorsal, anal and caudal fin) and occurrence of urinary calculi

Conditions	*n*	Lower jaw (%)	Upper jaw (%)	Anterior dorsal fin (%)	Posterior dorsal fin (%)	Anal fin (%)	Caudal fin (%)	Urinary calculi (%)
Control	3	25.0 ± 5.0^b^	10.0 ± 2.9	6.7 ± 1.7	40.0 ± 7.6	11.7 ± 1.7	31.7 ± 1.7	8.3 ± 1.7
LA 7.7	3	30.0 ± 5.8^b^	5.0 ± 2.9	5.0 ± 2.9	41.7 ± 6.0	11.7 ± 6.0	43.3 ± 8.8	3.3 ± 3.3
HA 7.5	3	6.7 ± 4.4^a^	11.7 ± 1.7	10.0 ± 2.9	53.3 ± 6.0	10.0 ± 2.9	40.0 ± 5.0	13.3 ± 4.4

Mean ± SEM; *n* is the number of individuals

The different letters indicate significant differences among conditions (*α* = 0.05)

### Gut development

The ratio of one pancreatic enzyme activity in intestinal segment to that enzyme activity in whole digestive tract is a marker of pancreatic secretion and degree of maturation. These ratios were not different among experimental conditions for either trypsin or amylase [ANOVAs, *F*(2,27) = 0.02, *P* = 0.98 and *F*(2,27) = 0.03, *P* = 0.96, respectively, Table 5].

**Table 5:** Digestive maturation index of the 45 days-post-hatching larvae in the three different experimental conditions: control condition ($P_{{{\text{CO}}_{2} }}$ = 590 µatm, pH total = 7.9), low acidification condition (LA 7.7, $P_{{{\text{CO}}_{2} }}$ = 980 µatm, pH total = 7.7), and high acidification condition (HA 7.5, $P_{{{\text{CO}}_{2} }}$ = 1520 µatm, pH total = 7.5)

Conditions	*n*	Trypsin (%)	Amylase (%)	Ratio AP·Leu-Ala^−1^ (×100)	Ratio AN·Leu-Ala^−1^ (×1000)
Control	9	46.79 ± 4.98	86.89 ± 0.99	0.75 ± 0.04	0.77 ± 0.02
LA 7.7	9	45.72 ± 2.95	87.01 ± 1.06	0.70 ± 0.05	0.77 ± 0.04
HA 7.5	9	47.04 ± 4.38	87.47 ± 1.00	0.83 ± 0.07	0.82 ± 0.04

Index are expressed as percentage of the secretion activity of released pancreatic enzymes (trypsin and amylase; percent activity in the intestinal segment related to total activity in the pancreatic and intestinal segment), and as relative activity of brush border membrane enzymes (alkaline phosphatase, AP, and aminopeptidase N, AN) related to the activity of the cytosolic enzyme (leucine–alanine peptidase, Leu-Ala)

Mean ± SEM; *n* is the number of individuals

Difference was not significant

The ratio representing the relative activity of the enzymes in the brush border membrane [alkaline phosphatase (AP), and aminopeptidase N (AN)] as a percentage of the activity of the cytosolic enzyme [leucine–alanine peptidase (Leu-Ala)] indicates the developmental status of the enterocytes. There were also no significant differences among experimental conditions for AP·Leu-Ala^−1^ and AN·Leu-Ala^−1^ ratios [ANOVAs, *F*(2,27) = 0.86, *P* = 0.46 and *F*(2,27) = 0.39, *P* = 0.69, respectively, Table 5].

### Response in gene expression

Microarray analysis: After applying the Benjamini–Hochberg-corrected threshold, no significant differences in transcripts levels were observed among fish reared in the different experimental conditions (ANOVAs, *P* > 0.10) (see Supplementary Table 1). The magnitude of the changes in gene transcription among conditions ranged from 3.03-fold under-transcription (with 10 genes having more than a 2.0-fold under-transcription, including one annotated gene involved in protein coding) to 5.76-fold over-transcription (with 20 genes having more than a 2.0-fold over-transcription including six annotated genes involved in protection against oxidative damage, immunity and inflammation response).

qPCR analysis: Expression data on the candidate genes revealed no difference among larvae from the different experimental conditions (Table 6). The transcript levels of osteocalcin [used as a marker of ossification process, ANOVA, *F*(2,90) = 1.76, *P* = 0.25] and CA2 [ANOVA, *F*(2,90) = 0.06, *P* = 0.94], CA4 [ANOVA, *F*(2,90) = 2.71, *P* = 0.14], Slc9a1 [ANOVA, *F*(2,90) = 1.55, *P* = 0.29], Slc9a3 [ANOVA, *F*(2,90) = 0.38, *P* = 0.70], Slc4a4 [ANOVA, *F*(2,90) = 2.82, *P* = 0.14] and Slc4a1 [ANOVA, *F*(2,90) = 1.09, *P* = 0.39], all used as markers of acid–base balance regulation, were not significantly influenced by the experimental conditions.

**Table 6:** Relative transcript levels of genes involved in bones mineralization (osteocalcin) and acid–base balance adjustments (CA2, CA4, Slc9a1, Slc9a3, Slc4a4, and Slc4a1) from whole sea bass larvae at 45 days-post-hatching in the three different experimental conditions: control condition ($P_{{{\text{CO}}_{2} }}$ = 590 µatm, pH total = 7.9), low acidification condition (LA 7.7, $P_{{{\text{CO}}_{2} }}$ = 980 µatm, pH total = 7.7), and high acidification condition (HA 7.5, $P_{{{\text{CO}}_{2} }}$ = 1520 µatm, pH total = 7.5), using GAPDH as housekeeping gene (relative expression in arbitrary units)

Conditions	*n*	Osteocalcin	CA2	CA4	Slc9a1	Slc9a3	Slc4a4	Slc4a1
Control	30	1.41 ± 0.11	1.07 ± 0.06	0.96 ± 0.06	1.04 ± 0.04	0.76 ± 0.05	1.17 ± 0.07	0.81 ± 0.06
LA 7.7	30	1.13 ± 0.08	1.10 ± 0.07	1.03 ± 0.09	1.12 ± 0.07	0.87 ± 0.07	1.07 ± 0.07	0.73 ± 0.05
HA 7.5	30	1.36 ± 0.12	1.08 ± 0.07	0.68 ± 0.06	0.89 ± 0.07	0.81 ± 0.06	0.90 ± 0.06	0.82 ± 0.07

Mean ± SEM; *n* is the number of individuals

Difference was not significant

## Discussion

The main objective of this study was to investigate, at various levels of biological organizations, the response of larval sea bass (*Dicentrarchus labrax*) to ocean acidification. We demonstrated that chronic exposure to predicted levels of ocean acidification had a limited influence on the survival and growth of the larvae from the low acidification condition. The most noticeable effect was an improvement of larvae skeletal development (faster mineralization and reduction in deformities), under the most severe hypercapnic condition tested. No adverse effect of water acidification on the digestive developmental process was observed. At the transcriptomic level, no significant difference among experimental treatments was observed in whole larvae, suggesting that the molecular pathways (even those involved in skeletogenesis or acid–base regulation) were not regulated at this scale. Our results therefore suggest that contemporary sea bass larvae will not need to mobilize specific defense mechanisms to be able to cope with projected ocean acidification conditions.

Chronic exposure to predicted ocean acidification conditions since two dph resulted in limited effects on the growth and survival of 45 dph sea bass larvae. Compared to previous studies under normocapnic conditions (survival ~45–68% and mass ~35–45 mg, Darias et al. 2010b; Vanderplancke et al. 2015), observed larval survival (49–62%) and mass (50–64 mg) at 45 dph was high under all experimental conditions. Fish from the low acidification condition yielded puzzling results as they displayed the highest survival rate combined with the lowest growth compared to the fish reared under the other conditions. The better survival and therefore higher density in the low acidification rearing tanks could have lead to density-dependent effects explaining, at least in part, the impaired growth observed (see also Zouiten et al. 2011). Nevertheless, our study highlighted overall good survival and growth of sea bass larvae under future acidification conditions. As the completion of the larval stage is crucial for fish population recruitment (reviewed in Houde 2008), this is an important observation. In the context of predicting the potential repercussions of ocean acidification, it is indeed crucial to anticipate the future of fish populations that have a current commercial interest.

The limited effect of predicted ocean acidification conditions on survival and growth have already been reported for both tropical (clownfish, *Amphiprion percula*, cobia, *Rachycentron canadum*) and temperate species (Baltic cod, *Gadus morhua*, walleye Pollock, *Theragra chalcogramma*) (Munday et al. 2009; Bignami et al. 2013; Frommel et al. 2013; Hurst et al. 2013). However, a reduction in survival (−48 to −74%) and growth (−10 to −30%) have also been reported in temperate species such as the inland silverside (*Menidia beryllina*), the summer flounder, (*Paralichthys dentatus*), and the Atlantic herring (*Clupea harengus*) (Baumann et al. 2012; Chambers et al. 2014; Frommel et al. 2014). This variability in the larval response may be explained partly by inter-species difference in larval acidification sensitivity. Another explanation may also be the difference in ontogenetic stage at which the exposition took place (egg, embryo, larvae) as the embryonic stage could be particularly sensitive to acidification, reducing larval survival (Baumann et al. 2012). The inconsistency among studies may also be explained by differences in experimental approaches, in particular in relation to the level ($P_{{{\text{CO}}_{2} }}$ from 775 to 1800 µatm) and duration of acidification exposure (from 28 to 39 days). It may also be attributable to the origin of the parental population (wild vs domesticated) which may have experienced different $P_{{{\text{CO}}_{2} }}$ conditions. Farmed fish are liable to be exposed to hypercapnic conditions which can favor resistance to water acidification.

Faster larval skeletal development was observed under the most severe acidification condition. Fish are internally calcifying organisms, and their capacity to maintain their acid–base balance was initially believed to protect them from the consequences of environmental acidification. Yet, experiments have shown that otolith growth may be affected by future acidification conditions. In white sea bass (*Atractoscion nobilis;* Checkley et al. 2009), clownfish (*Amphiprion percula;* Munday et al. 2011b), and cobia (*Rachycentron canadum*; Bignami et al. 2013), otolith length and area have been observed to increase under hypercapnia ($P_{{{\text{CO}}_{2} }}$ 794–1721 µatm). Only one study examined skeleton development in fish (spiny damselfish *Acanthochromis polyacanthus*) under future ocean acidification ($P_{{{\text{CO}}_{2} }}$ 850 µatm, pH 7.8), and no effect was observed (Munday et al. 2011a). In this latter study, although the size of specific skeletal elements such as the width and length of the fins, spine, and vertebra was measured, no evaluation of the skeleton mineralization status was conducted. The present study is thus the first to highlight larval skeletal ossification taking place earlier under projected acidification conditions.

The mechanisms potentially linking water CO_2_ content and skeleton mineralization are not clear. It is well known that to regulate extracellular and intracellular pH in the face of hypercapnia, fish elevate plasma bicarbonate concentration (Esbaugh et al. 2012; reviewed in Heuer and Grosell 2014). Following research on the otolith (Checkley et al. 2009; Munday et al. 2011b), it could be hypothesized that the accumulation of bicarbonate in the internal fluids is liable to favor the mineralization of the skeleton by enhancing calcium precipitation on the bone matrix. It has also been proposed that additional buffering of tissue pH with non-bicarbonate ions under acidified conditions could interfere with larval skeletal development (Munday et al. 2011a). This mechanism still needs to be elucidated. Nonetheless, our transcriptomic analyses showed that the expression pattern of osteocalcin, a marker of bone differentiation and mineralization (Mazurais et al. 2008; Darias et al. 2010a), was not affected by ambient $P_{{{\text{CO}}_{2} }}$. This suggests that the observed faster skeletogenesis may be an indirect effect of internal pH homeostasis, rather than the result of a specific calcification regulatory pathway.

The faster mineralization process was accompanied with a lower occurrence of skeletal deformities, both macroscopically evident and lower jaw deformities. Few studies have examined the impact of future ocean acidification on the occurrence of larval deformities. However, those to have done so have highlighted increased occurrence in larval *Solea senegalensis* (Pimentel et al. 2014) and *Paralichthys dentatus* (Chambers et al. 2014). These authors hypothesized that this result could arise from water acidification affecting the metamorphosis itself, i.e., affecting the migration process of the craniofacial region (Chambers et al. 2014). It has also been suggested that these deformities could, in part, be linked to the rearing conditions (Pimentel et al. 2014). Intensive rearing, stress, or inappropriate tank hydrodynamism has indeed been associated with increased occurrence of deformities (Boglione et al. 2013). A number of studies have also reported that the occurrence of skeletal deformities could be linked to growth rate. The temperature-driven increase in larval growth rate has been associated with morphological deformities during fish development (Georgakopoulou et al. 2010). In the present case, the reduction in the occurrence of deformities observed in fish from the most severe acidification condition cannot be attributed to differences in growth rate as these fish displayed similar growth than the control fish. As the skeletal shape of larvae is critical for their swimming capacities, ability to disperse or feed and, ultimately, for their survival (Koumoundouros et al. 2001; Boglione et al. 2013), our results suggest that some beneficial repercussions on the larval recruitment could be expected for sea bass in future conditions.

Based on several indices of maturation of the digestive tract, at both the pancreatic and intestinal levels, the present study did not reveal adverse effects of acidification on the digestive developmental process. Studies that examined larval development through the maturation of this specific function are still scarce and only one used similar proxies to ours (Pimentel et al. 2015). These authors reported normal ontogenetic development under acidification conditions ($P_{{{\text{CO}}_{2} }}$: 1600 µatm, pH 7.5), even if a general decrease in enzymatic activities was observed. These maturation proxies are good indicators of possible repercussions on the juvenile and adult digestive function (reviewed in Zambonino-Infante and Cahu 2001). The absence of difference among experimental conditions suggests that the sea bass larvae follow a normal development reaching normal juvenile and adult mode of digestion.

Using a transcriptomic integrative approach, we investigated a wide range of physiological processes potentially regulated following exposure to future acidification conditions. Contrary to our expectation, experimental data did not reveal any significant difference among the conditions. This result suggests that if some physiological regulations occurred in response to the environmental conditions, in particular those related to internal acid–base balance, they did not translate into changes at the global transcriptomic level. Accordingly, a previous study on Atlantic herring (*Clupea harengus*) found that the proteome was also resilient to future $P_{{{\text{CO}}_{2} }}$ conditions (1800 µatm, Maneja et al. 2014). Integrative transcriptomic analyses on calcifying organisms revealed an up-regulation of genes involved in ions transport and CO_2_ hydration, likely to facilitate acid–base compensation (Evans et al. 2013; Padilla-Gamino et al. 2013). To address more specifically the acid–base response in fish, several genes (CA2, CA4, Slc9a1, Slc9a3, Slc4a4, Slc4a1) involved in acid–base homeostasis (Tseng et al. 2013; reviewed in Heuer and Grosell 2014) were investigated using qPCR. This analysis was consistent with the integrative approach as it did not reveal difference in gene expression among experimental conditions. However, it should be noted that the entire larvae was used for the RNA extraction and that this may hide tissue-specific responses to acidification. Another possibility could be that the regulation needed to face future conditions might have occurred before the 45 dph, as mRNA abundance can be very dynamic in developing organisms (reviewed in Mazurais et al. 2011). By that time, the regulatory processes and protein abundance may have reached equilibrium.

The absence of regulation of the genes involved in acid–base homeostasis contradicts previous results that suggested ocean acidification has an impact on these regulatory processes (Tseng et al. 2013; reviewed in Heuer and Grosell 2014). When acutely exposed to ocean acidification (hours to few days), it has been reported that teleost larvae maintain their internal acid–base balance through regulatory processes which involve the combined action of co-transporters at the gills (NA^+^/H^+^ exchanger, NA^+^/HCO_3_^−^ co-transporter, HCO_3_^−^ transporter) as well as bicarbonate uptake (reviewed in Melzner et al. 2009; Esbaugh et al. 2012; Heuer and Grosell 2014). In the present study, after 45 days of exposure, the genes involved in ions co-transport were not differentially expressed among the conditions. This result suggests that the 45 dph larvae have been able to cope with the future conditions without having to rely on regulatory processes at that time. One explanation could be that the adjustments required were under the triggering threshold of gene expression regulation. The activity of protein and transporters currently involved in acid–base homeostasis of the larvae could be sufficient to reach the adjustment need of the projected acidification levels.

The tolerance of sea bass larvae to projected levels of ocean acidification may be partially explained by the wide range of habitats this species occupies during its life cycle. The European sea bass has a large distribution area and inhabits a broad range of habitats from offshore (as larvae) to inshore nurseries to coastal zones as adult (Jennings and Pawson 1992). Young sea bass experience strong environmental fluctuations that characterize coastal environments, such as wide qualitative and quantitative fluctuations in food supply, salinity, dissolved oxygen, and even $P_{{{\text{CO}}_{2} }}$ and pH due to water hydrodynamism and eutrophication (Cai et al. 2011; Mauduit et al. 2016). The use of such fluctuating habitats may constitute a factor that permitted the evolution of plasticity of sea bass larvae (Melzner et al. 2009; Bignami et al. 2013; Tseng et al. 2013). This broad range of conditions may have contributed to enlarge the physiological toolbox of the sea bass, allowing the larvae to better cope with environmental constraints including hypercapnia and related water acidification. Fish species adapted to less variable conditions in their natural environments may be more sensitive to $P_{{{\text{CO}}_{2} }}$ constraints (reviewed in Pörtner et al. 2004; Munday et al. 2008; Melzner et al. 2009). Considering the rate of ocean acidification, fish species that already possess such adaptive physiological toolbox may have better chance to prosper.

The present study examined several physiological parameters linked to larvae survival, growth, and development and implemented analytical approaches with and without a priori hypotheses. It revealed that sea bass larvae can cope with acidification scenarios predicted to occur within the next century. However, this does not exclude the possibility that other effects, not measured or detected at the larval stage, could be revealed at later life stages, or even across generations. Previous studies have already reported that exposure to hypoxia during early life stage can have long-lasting effects in sea bass (Vanderplancke et al. 2015) as well as transgenerational effects in zebrafish, *Danio rerio* (Ho and Burggren 2012). Additional research on the long-term and transgenerational effect of ocean acidification could be highly valuable to better understand the global effect of ocean acidification on populations. Moreover, global climate change has to be considered as a multidimensional issue. Interactions between increased $P_{{{\text{CO}}_{2} }}$ and other environmental factors such as water temperature or hypoxia levels are expected and could be even more challenging through synergetic effects that could exceed the functional thresholds of key physiological processes. Further investigations on the interactive effect of different stressors should therefore be promoted.

## Electronic supplementary material

Below is the link to the electronic supplementary material.

Supplementary material 1 (PDF 24 kb)
