# Supplementary material for: The development of contemporary European sea bass larvae (Dicentrarchus labrax) is not affected by projected ocean acidification scenarios
Source: Mar Biol. 2017 Jun 29;164(7):155. doi: 10.1007/s00227-017-3178-x (PMC5491573; doi:10.1007/s00227-017-3178-x)
Supplement: Supplementary file 1 — Supplementary material 1 (PDF 24 kb) [file 227_2017_3178_MOESM1_ESM.pdf]

The development of contemporary European sea bass larvae (*Dicentrarchus labrax*) is not affected by projected ocean acidification scenarios

Amélie Crespel 1, José-Luis Zambonino-Infante 1, David Mazurais 1, George Koumoundouros 3, Stefanos Fragkoulis 3, Patrick Quazuguel 1, Christine Huelvan 1, Laurianne Madec 1, Arianna Servili 1, Guy Claireaux 2

1Ifremer, Laboratoire Adaptation, Reproduction et Nutrition des poissons, LEMAR (UMR 6539)29280PlouzanéFrance

2Université de Bretagne Occidentale, LEMAR, (UMR 6539), Laboratoire Adaptation, Reproduction et Nutrition des poissons29280PlouzanéFrance

3Biology DepartmentUniversity of CreteVasilika Vouton70013HeraklioCreteGreece

Table S1 Genes differential transcription according to the experimental conditions: C, control condition (P_{CO2} = 590 µatm, pH total = 7.9); LA, low acidification condition (P_{CO2} = 980 µatm, pH total = 7.7); and HA, high acidification condition (P_{CO2} = 1520 µatm, pH total = 7.5). *P* values were obtained after applying Benjamini-Hochberg corrected threshold. Difference was not significant

Probe	Description	Function	*P* values	Fold change C - LA	Fold change C - HA
	Under-transcription				
DLPD01687_1	?		0.119	-1.593	-3.036
DLPD01687_2	?		0.119	-1.366	-2.878

DLPD04778_1	?		0.100	-1.277	-2.772
DLPD04778_2	?		0.100	-1.298	-2.737
DLPD17752_2	?		0.108	1.483	-2.672
DLPD15791_1	?		0.154	-1.716	-2.223
DLPD05123_1	N(alpha)-acetyltransferase 20, NatB catalytic subunit	protein coding	0.119	-2.631	-2.148
DLPD17404_1	?		0.136	-1.320	-2.069
DLPD17404_2	?		0.140	-1.365	-2.042
DLPD04843_1	zgc:123297		0.190	-1.241	-2.037
	Over-transcription				
DLPD01915_2	?		0.100	2.762	5.766
DLPD01915_1	?		0.100	2.651	5.631
DLPD05835_1	major histocompatibility complex class I UBA	immune response	0.100	3.771	5.547
DLPD01686_1	?		0.100	1.971	3.469
DLPD05835_2	major histocompatibility complex class I UBA	immune response	0.100	2.958	3.003
DLPD00118_2	?		0.100	4.165	2.966

DLPD07034_2	?		0.154	1.469	2.547
DLPD17151_2	?		0.100	1.159	2.534
DLPD17151_1	?		0.119	1.180	2.374
DLPD07945_2	?		0.100	1.558	2.294
DLPD18549_1	?		0.150	1.162	2.259
DLPD07035_1	?		0.162	1.365	2.208
DLPD08598_1	?		0.162	1.322	2.177
DLPD09474_1	hemopexin	Oxidative damage protection	0.154	1.459	2.099
DLPD07035_2	?		0.168	1.337	2.086
DLPD07034_1	?		0.168	1.272	2.080
DLPD17643_1	C-reactive protein 1	Inflammation response	0.119	-1.437	2.042
DLPD09474_2	hemopexin	Oxidative damage protection	0.154	1.459	2.042
DLPD17643_2	C-reactive protein 1	Inflammation response	0.119	-1.432	2.017
DLPD04465_2	ribosomal protein L17		0.119	2.531	1.531
